# Physiological and nutritional stress response of African elephants within the lantana-dominated Lower Imenti Forest Reserve in Kenya

**DOI:** 10.1093/conphys/coaf060

**Published:** 2025-08-12

**Authors:** Sandy Oduor, Isaac Lekolool, Mathew Mutinda Ndunda, Sharon Mulindi, Jeremiah Poghon Kaitopok, Susie Weeks, Enock Ochieng, Janine L Brown, Suzan Murray, Jenna M Parker, Festus Ihwagi, Frank Pope, Linus Kariuki, Francis Gakuya, Charles Musyoki, George Wittemyer

**Affiliations:** Department of Biology, University of Nairobi, PO Box 30197-00100, Nairobi, Kenya; Department of Reproductive Biology, Smithsonian Conservation Biology Institute, 1500 Remount Road, Front Royal, VA 22630, USA; Save the Elephants, Marula Manor, Marula Lane, Karen, PO Box 54667-00200, Nairobi, Kenya; Wildlife Community Service Directorate, Kenya Wildlife Service, PO Box 40241-00100, Nairobi, Kenya; Wildlife Community Service Directorate, Kenya Wildlife Service, PO Box 40241-00100, Nairobi, Kenya; Wildlife Community Service Directorate, Kenya Wildlife Service, PO Box 40241-00100, Nairobi, Kenya; Wildlife Community Service Directorate, Kenya Wildlife Service, PO Box 40241-00100, Nairobi, Kenya; Mount Kenya Trust, PO Box 690-10400, Nanyuki, Kenya; Mount Kenya Trust, PO Box 690-10400, Nanyuki, Kenya; Center for Species Survival, Smithsonian Conservation Biology Institute, 1500 Remount Road, Front Royal, VA 22630, USA; Global Health Program, Smithsonian Conservation Biology Institute, Washington, DC, USA; School of Natural Resources, Lake Superior State University, Sault Ste. Marie, MI 49783, USA; Save the Elephants, Marula Manor, Marula Lane, Karen, PO Box 54667-00200, Nairobi, Kenya; Save the Elephants, Marula Manor, Marula Lane, Karen, PO Box 54667-00200, Nairobi, Kenya; Wildlife Community Service Directorate, Kenya Wildlife Service, PO Box 40241-00100, Nairobi, Kenya; Wildlife Health and Laboratories, Wildlife Research & Training Institute, PO Box 842-20117, Naivasha, Kenya; Wildlife Community Service Directorate, Kenya Wildlife Service, PO Box 40241-00100, Nairobi, Kenya; Save the Elephants, Marula Manor, Marula Lane, Karen, PO Box 54667-00200, Nairobi, Kenya; Department of Fish, Wildlife, and Conservation Biology, Colorado State University, 1474 Campus Delivery, Fort Collins, CO, USA

**Keywords:** Faecal glucocorticoid metabolites, faecal thyroid metabolites, habitat degradation, invasive species

## Abstract

Invasive species can alter the ecology of protected areas, substantially lowering the habitat quality for vertebrate communities. The Lower Imenti Forest on Mt. Kenya’s northeastern slope has experienced habitat disturbance, degrading the system and resulting in the establishment of invasive species, including lantana (*Lantana camara*), throughout the area. Following reports of high mortality and poor conditions among the African savanna elephants (*Loxodonta africana*) inhabiting the area, we assessed the status of two endocrine indicators of their physiological condition. Specifically, we assessed the physiological stress response by measuring faecal glucocorticoid metabolites (fGCM) and the nutritional stress response by measuring faecal thyroid (fT3) concentrations in elephant faecal samples collected in the forest. To better interpret the hormone levels, we compared the hormone concentrations in the Imenti faecal samples to concentrations from reference levels indicative of extreme nutritional stress (from faecal samples of elephants experiencing drought-induced mortality) and adrenal stress (from elephants experiencing high levels of human–elephant conflict). The concentrations of fT3, a biomarker of nutritional stress response, found in elephant faecal samples from the Lower Imenti Forest were lower than the drought-stressed reference levels, suggesting lower levels of energy intake and assimilation of forage resources in elephants from this area. The concentration of fGCM, a biomarker of physiological stress response, was higher than the human–elephant conflict reference levels, suggesting the elephants in Lower Imenti were experiencing a higher physiological stress response. We found no differences between fT3 and fGCM concentrations in samples assigned to different age classes (juvenile, subadults, adults), suggesting the physiological problems were not age specific. Findings from our physiological study suggest that restricted movement and reduced forage availability due to lantana infestation in the Lower Imenti Forest may be driving the elevated nutritional stress, potentially contributing to the concerning mortality observed in the area. We discuss the use of endocrine markers to ascertain wildlife responses to degraded habitats.

## Introduction

Natural habitats for many wildlife species have already been reduced by 18% and are projected to be reduced by up to 23% by the year 2100 due to continued habitat degradation and loss (Monastersky, 2014; Beyer and Manica, 2020). A key correlate of habitat degradation and loss is colonization by invasive alien species, which alters ecosystem structure and functioning, leading to biotic impoverishment and homogenization (Foxcroft *et al.*, 2010; Stotz *et al.*, 2019). Monitoring wildlife populations in the face of altered habitat quality can provide ecological and conservation-relevant insights into the status of populations of concern and thresholds of degradation that are problematic for species’ well-being (Wikelski and Cooke, 2006; Angelier and Wingfield, 2013; Madliger and Love, 2016).

Invasive species negatively affect the physical attributes of the ecosystem, such as soil nutrients, abundance and richness of native species and ecosystem functioning, such as carbon cycling (Blackburn *et al.*, 2011; Ramaswami and Sukumar, 2016; Davidson *et al.*, 2018; Wekhanya *et al.*, 2020). One notable invasive weed is the *Lantana camara*, primarily native to the American tropics (Kato-Noguchi and Kurniadie, 2021). It is an evergreen perennial woody shrub that grows in open, unshaded environments and becomes dominant in degraded or frequently burned areas (Safari and Byarugaba, 2008; Day and Zalucki, 2009; Fischer *et al.*, 2010; Kipkoech *et al.*, 2020). It has a persistent seed bank and forms a dense thicket, making it difficult to eradicate (Ssali *et al.*, 2024). While the ecological impact of *L. camara* on wild animals is well-documented, particularly its hepatotoxic effects (Sharma *et al.*, 2007) and its negative influence on foraging behaviour and habitat use (Wilson *et al.*, 2013; Wilson *et al.*, 2014), broader research on invasive species further highlights their role in poisoning wildlife and suppressing native forage plants and recruitment of indigenous forest species through allelopathy (Day and Zalucki, 2009; Wilson *et al.*, 2014; Rastogi *et al.*, 2023). However, the physiological response of wild animals to such habitat degradation remains relatively understudied. Physiological monitoring offers valuable information regarding the status of an animal as they are increasingly exposed to varying habitat alterations and degradation (Wingfield and Kitaysky, 2002).

One of the primary physiological indicators used to monitor the effects of habitat change on an animal population is the concentrations of glucocorticoid (GC) hormones, given their role in energy metabolism and involvement in normal physiological function (Boonstra, 2013). Stressful experience in vertebrates triggers activation of the hypothalamic–pituitary–adrenal axis, resulting in the release of GC in response to an external ‘stressor’, which helps an animal cope with that external stressor (Dantzer *et al.*, 2014; MacDougall-Shackleton *et al.*, 2019). Prolonged elevation of GCs within the bloodstream can negatively affect animal physiology in many ways, including suppressing immune function, leading to increased disease susceptibility, decreased wound healing, inhibition of reproduction and decreased growth (Sapolsky *et al.*, 2000; Busch and Hayward, 2009; Romero and Wingfield, 2015), all of which can affect fitness. Another physiological indicator is the concentrations of thyroid hormones, which function to increase basal metabolic rates, stimulate protein synthesis and increase glucose availability to cells (Pasciu *et al.*, 2022; Pasciu *et al.*, 2024). Thyroid hormones are activated by the hypothalamic–pituitary–thyroid axis, leading to the production of triiodothyronine (T3) and tetraiodothyronine (T4) from thyroid follicles (Behringer *et al.*, 2018). T3 is commonly considered more biologically active and potent than T4 and therefore has greater biological and clinical importance, while T4 is a prohormone and stored in the body longer and not released as acutely, and thus is less informative (Ingbar and Braverman, 1975; Fisher and Polk, 1989; Hulbert, 2000). T3 decreases during periods of food restriction, and hence, lowered concentrations reflect resource limitations (Gobush *et al.*, 2014), making it a useful physiological biomarker for assessing whether wildlife are obtaining adequate nutrition (Wasser *et al.*, 2010). In elephants, both GC and T3 hormones can be measured noninvasively as metabolites in faeces (i.e. fGCM and fT3) and reflect the hormonal state about 36 h preceding defecation (Wasser *et al.*, 2000).

Habitat fragmentation and degradation expose species to unpredictable environmental stressors, such as climate extremes and nutritional deficiencies, which can negatively affect native vertebrate populations (Fischer and Lindenmayer, 2007; Janin *et al.*, 2011; Haddad *et al.*, 2015). Indeed, studies have documented higher GCs and poor body condition in wildlife from disturbed habitats (Romero, 2004; Ellis *et al.*, 2012) as well as altered space use and altered habitat preference (Jachowski *et al.*, 2012). In African savanna elephants (*Loxodonta africana*), habitat degradation has been linked to high mortality rates due to droughts in the Tsavo ecosystem (Wato *et al.*, 2016), climate change-driven cyanobacteria blooms or bacterial infection and resultant septicemia (van Aarde *et al.*, 2021; Veerman *et al.*, 2022; Foggin *et al.*, 2023) and fires (Woolley *et al.*, 2008), highlighting the demographic impacts of habitat change on elephants.

Recent reports of African savanna elephant deaths in the Lower Imenti Forest of Kenya have raised conservation concerns regarding the health and welfare of the remaining elephants in the area. Autopsy reports from 24 carcasses recorded between January and November 2023 revealed tick infestations and anaemia (Kariuki *et al.*, 2023), suggesting potential issues with the immunity and nutritional status of the population (Martin *et al.*, 2010). These mortality events occurred shortly after the electric fencing of the forest in an effort to reduce human–elephant conflict in the area. Physiological assessment of these elephants can provide insight into the likely drivers of their compromised health. This is the first study to investigate the endocrine status of elephants in a habitat degraded by invasive species.

In this study, we assessed the physiological (fGCM) and nutritional (fT3) status of African elephants within the Lower Imenti Forest, an area that has progressively experienced major changes in vegetation types over the past several decades due primarily to pervasive *L. camara* invasion (Chebet, 2011). To better interpret our results, we compared hormone concentrations from the Imenti elephants to index levels of high fGCM concentrations, from elephants experiencing high levels of human–elephant conflict, and low fT3 concentrations, from elephants experiencing severe drought. All index levels were derived from elephants in the greater Laikipia/Samburu ecosystems, of which the Imenti forest is a part. Specifically, we tested the hypothesis that elephants in the Lower Imenti Forest (an area experiencing habitat degradation due to the proliferation of *L. camara*) would exhibit reduced energy intake due to habitat conditions (Wilson *et al.*, 2014). Accordingly, we predicted that the elephants within the Lower Imenti Forest would have lower fT3 concentrations, indicating a poorer diet, than our studied elephants in the greater ecosystem. This is because reduced energy intake (i.e. resource limitation) has been shown to decrease fT3 levels, while increased energy intake leads to higher fT3 levels (Gobush *et al.*, 2014; Schaebs *et al.*, 2016; Behringer *et al.*, 2018). Similarly, if the studied elephants were experiencing higher stressors due to confinement and the adverse ecological effects of the degraded habitat, we would expect to observe higher fGCM concentrations than index values (Jachowski *et al.*, 2012). We discuss our findings in the context of the management problems facing the lantana-dominated Lower Imenti Forest and restricted elephant movement. Furthermore, we discuss how our findings highlight the value of endocrine assessments to evaluate the physiological state of elephants in management situations of concern.

## Materials and Methods

### Ethical approval

Permission to conduct this study was granted by the Wildlife Research and Training Institute (Permit No. WRTI-0110-11-21) and the National Commission for Science, Technology & Innovation (Permit No. NACOSTI/P/21/4067).

### Study site

**Figure 1:**
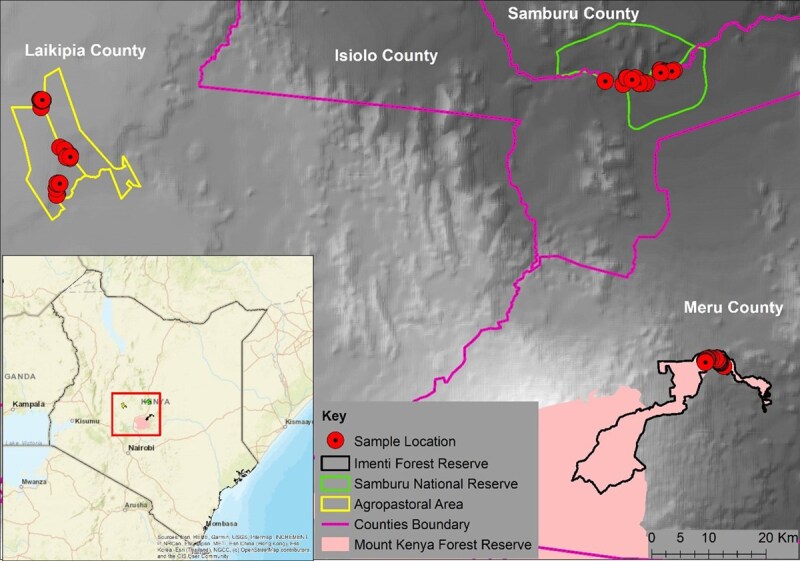
A map of the study area showing the locations of samples collected within Lower Imenti Forest Reserve, Samburu National Reserve and agropastoral areas.

The study was carried out in the Lower Imenti Forest (Fig. 1) of Meru County, Kenya, and managed by the Kenya Forest Service (KFS). The forest is an extension of the Mt. Kenya forest ecosystem, covering an area of 12 888 hectares and a migratory corridor for elephants in transit between Mt. Kenya National Park and the northern grazing area (Wass, 1995). The forest has a variety of indigenous and exotic trees, including Meru oak (*Vitex keniensis*), podo (*Podocarpus milanjianus*), red skin wood (*Pygeum africanum*), camphor (*Ocotea usambarensis*) and Cypress (*Cupressus lusitanica*). Due to short-term degazetting of its protected status during which slash and burn agriculture occurred throughout the forest, the forest is degraded and experienced subsequent invasion by *L. camara,* which has expanded rapidly to subsume much of the forest (KWS, 2000). The forest section is a known staging ground for crop-raiding elephants (Cerling *et al.*, 2009) and, due to the resulting rampant human–elephant conflict, a 60-km solar-powered electric fence was constructed to mitigate conflict in the area. As part of this fence, a one-way gate was constructed in the Lower Imenti Forest to allow elephants to enter the forest but prevent them from exiting, utilizing sensors that detect the presence of a large mass at the gate (Ikime *et al.*, 2023). After the construction of the fence, elephants were unable to enter the neighbouring agricultural areas, which coincided with reports of elephants in poor physical condition and experiencing tick infestation and high mortality (Kariuki *et al.*, 2023).

### Faecal sample collection

We collected 34 fresh faecal samples in the Lower Imenti Forest between 1 and 7 March 2024 at the end of a typical dry season. A researcher in the company of forest reserve rangers searched for elephants following the paths of least resistance and likely used by elephants based on the fresh elephant trails. We located fresh dung samples by following the direction of elephant sounds in the forest (e.g. rumbles, breaking branches). At each location where signs were heard, we collected moist faecal samples that were warm, had a strong odour and showed signs of recent urination. This sampling method aligns with previous approaches for assessing sample freshness. To prevent sampling the same individual, we collected dung of different circumference at any given location. For each sample, we recorded its GPS location and assigned the sample to elephant age groups (juvenile = 0–8 years; subadult = 9–17 years; adult ≥ 18 years) based on boli (dung) circumference following established criteria (Morrison *et al.*, 2005). Categorization of sex was not possible because the forest was too dense to see who had produced each dung sample, and we did not have access to genetic sequencing. Approximately 200 g of faecal sample from each individual was placed in a Whirl-Pak bag (Nasco), given a unique identification code, and placed in a cooler box with ice packs in the field before being transferred to a −20°C freezer.

To contextualize our findings, we compared the samples collected from the Lower Imenti Forest with those collected during a time of severe drought from March to October 2022 in the Samburu National Reserve (*n* = 32). Samples from an agropastoral landscape (*n* = 50) were collected in the dry season from March to October 2022, when there was a drought, but not as severe as that observed in the more northern Samburu National Reserve Ecosystem and previously reported by Oduor *et al.* (2024). It is noted that both the Samburu National Reserve and the agropastoral study areas have not been invaded by the invasive *L. camara* (Table 1). Elephants within the Samburu National Reserve and the agropastoral areas were identified using catalogue recognition files, used by the NGO’s Save the Elephants in the national reserve (Wittemyer, 2001) and Space for Giants within the agropastoral area (Graham *et al.*, 2010). To avoid autocorrelation, each family group was visited no more than once, with samples collected from each individual no more than once. For individuals who could not be identified, the circumference of the dung was used to assign the elephants to an age group following established criteria (Morrison *et al.*, 2005). Table S1 summarizes the three study sites where samples were collected.

**Table 1:** A summary of elephant faecal samples collected across different age groups in the three compared study areas: Lower Imenti Forest Reserve, Samburu National Reserve and agropastoral areas

	Samples collected across different sites		
Age group	Imenti forest	Samburu reserve	Agropastoral
Juvenile	8	9	15
Subadult	10	10	18
Adult	16	16	17
Total	34	35	50

### Faecal sample processing and analysis

Hormones were extracted using an established wet-weight vortexing method (Edwards *et al.*, 2016) at the Endocrinology Laboratory of Mpala Research Centre in Kenya. First, the samples were thawed, thoroughly mixed and 0.5 g (±0.02) extracted by vortexing in 5 ml of 90% methanol in 16 × 125-mm glass tubes for 30 min, followed by centrifuging at 2500 rpm for 20 min. The resulting supernatants were decanted in another glass tube, dried in a warm water bath and reconstituted with 1 ml of assay buffer (Cat. No. X065; Arbor Assays, Ann Arbor, MI, USA). The samples were then sonicated until completely resuspended and stored at −20°C until analysis. Concentrations of fGCM and fT3 were measured by enzyme immunoassay (EIA) (DetectX^®^ Corticosterone EIA K014 and DetectX^®^ Triiodothyronine EIA K056, Arbor Assays) as described by Oduor *et al.* (2024). Intra- and interassay coefficients of variation for both corticosterone and T3 were maintained at <10% and <15%, respectively, and duplicates over 10% were reanalysed. Faecal extracts were diluted to 1:16 and 1:20 for GC metabolites and T3, respectively.

### Statistical analysis

Hormonal data for both fGCM and fT3 concentrations were log_10_ transformed to achieve normality. We then constructed multiple linear regression models for which the response variable was the log-transformed fGCM or fT3 concentrations. In both models, we included the effects of the study site and age group. The interaction term study site × age group was not significant and was removed from all models to facilitate interpretation of the main effects (Engqvist, 2005). The model was validated graphically by assessing for normality and heteroscedasticity (Zuur *et al.*, 2009). We compared the mean concentrations expressed as ±standard deviation (SD) of fGCM and fT3 across different study sites and age groups using one-way analysis of variance (ANOVA) and applied Tukey HSD for pairwise comparison of the significant effects across age groups and study sites. Tukey HSD was chosen because it is a widely accepted method for multiple pairwise comparisons that controls for Type I error across all tests, making it an appropriate statistical framework for our experimental design. All statistical analyses were performed in the statistical programme R version 4.4.0 (R Development Core Team, 2024).

**Figure 2:**
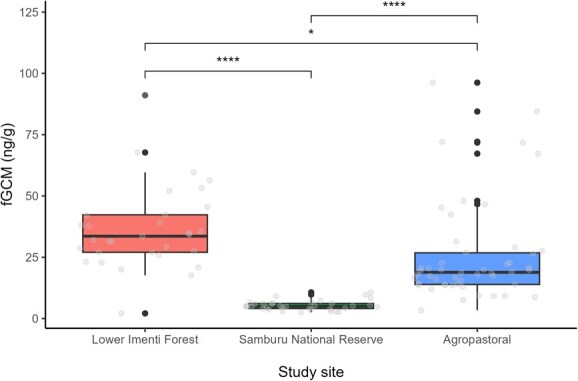
Influence of study site on faecal GC metabolite (fGCM) concentrations. Boxplots show the interquartile range with median lines. Grey dots indicate the fitted data points, while black dots above the box plots signify outliers. Statistical comparisons were conducted using one-way ANOVA, followed by *t* tests for comparison between sites. Asterisks denote significance: ^*^P ≤ 0.05, ^**^P ≤ 0.01, ^***^P ≤ 0.001, ^****^P ≤ 0.0001.

**Table 2:** Model estimates showing variation of fGCM concentrations in African elephants according to different predictor variables

Model: log(fGCM) ~ habitat location + age group					
Predictors	Estimates	SE	CI	Statistic	*P* value
Intercept	3.43	0.11	3.21 to 3.64	31.70	**<0.001**
Study site (Samburu National Reserve)	−1.86	0.14	−2.14 to −1.57	−13.03	**<0.001**
Study site (Agropastoral)	−0.45	0.14	−0.72 to −0.19	−3.36	**0.001**
Age group (juvenile)	−0.07	0.14	−0.34 to 0.21	−0.49	0.623
Age group (subadult)	0.19	0.13	−0.06 to 0.44	1.54	0.125
Observations	116				
*R* ^2^/*R*^2^ adjusted	0.65/0.64				

The reference category for the study site is the Lower Imenti Forest, while the reference category for the age group is adult. Variables that were statistically significant were indicated in bold.

## Results

The concentrations of fGCM (*n* = 116) averaged (±SD) 22.43 ± 19.98 ng/g and ranged from 2.11 to 96.22 ng/g. The concentrations of fT3 (*n* = 116) averaged 188.73 ± 276.42 ng/g and ranged from 1.39 to 1225.35 ng/g. The concentrations of fGCM for juveniles (17.39 ± 17.91 ng/g, *n* = 27) were lower compared to those of subadults (26.20 ± 22.90 ng/g, *n* = 35) or adults (22.50 ± 18.72 ng/g, *n* = 54), although these differences were not significant (ANOVA: *F*_2,113_ = 1.49, *P* = 0.23). The concentrations of fT3 for juveniles (246.53 ± 303.12 ng/g, *n* = 27) were higher compared to those of subadult (224.82 ± 321.91 ng/g, *n* = 35) or adults (136.44 ± 221.11 ng/g, *n* = 54), although these differences were not significant (ANOVA: *F*_2,113_ = 1.88, *P* = 0.16).

**Figure 3:**
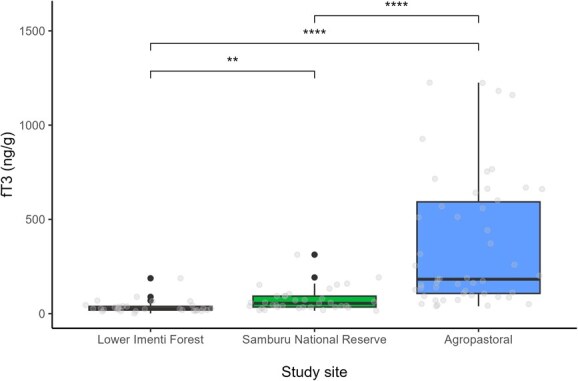
Influence of study site on faecal triiodothyronine (fT3) concentrations. Boxplots show the interquartile range with median lines. Grey dots indicate the fitted data points, while black dots above the box plots signify outliers. Statistical comparisons were conducted using one-way ANOVA, followed by *t* tests for comparison between sites. Asterisks denote significance: ^*^P ≤ 0.05, ^**^P ≤ 0.01, ^***^P ≤ 0.001, ^****^P ≤ 0.0001.

Our model for fGCM concentrations consisted of study site and age group (*F*_4,111_ = 51.35, *P* < 0.05, *R*^2^ = 0.64). Concentrations of fGCM differed across the three study sites. Elephants within the Samburu National Reserve (coefficient estimate = −1.86, 95% confidence interval [CI] = −2.14 to −1.57) and agropastoral study site (−0.45, 95% CI = −0.72 to −0.19) had lower fGCM concentrations relative to the reference category of the Lower Imenti Forest site (Fig. 2). A post hoc Tukey HSD test indicated that individuals within the Lower Imenti Forest had significantly higher fGCM concentrations than those within the Samburu National Reserve (*P* < 0.05) or agropastoral areas (*P* < 0.05). Additionally, individuals within agropastoral areas had significantly higher fGCM concentrations compared to individuals within the Samburu National Reserve (*P* < 0.05). Concentrations of fGCM estimated for juveniles (−0.07, 95% CI = −0.34 to 0.21) and subadult (0.19, 95% CI = −0.06 to 0.44) did not significantly differ relative to adults (Table 2). Our model for fT3 concentrations also included the study site and age group (*F*_4,111_ = 30.83, *P* < 0.05, *R*^2^ = 0.51). Concentrations of fT3 differed across the three study sites. Elephants within Samburu National Reserve (0.70, 95% CI = 0.26–1.14) and the agropastoral study site (2.07, 95% CI = 1.65–2.49) had higher fT3 concentrations relative to the reference category of the Lower Imenti Forest site (Fig. 3). A post hoc Tukey HSD test indicated that individuals within the Lower Imenti Forest had significantly lower fT3 concentrations than those within the Samburu National Reserve (*P* < 0.05) or agropastoral areas (*P* < 0.05). Additionally, individuals within agropastoral areas had significantly higher fT3 concentrations compared to individuals within the Samburu National Reserve (*P* < 0.05). Concentrations estimated for juveniles (0.23, 95% CI = −0.20 to 0.66) and subadult (0.16, 95% CI = −0.23 to 0.55) did not significantly differ relative to adults (Table 3).

**Table 3:** Model estimates showing variation of fT3 concentrations in African elephants according to different predictor variables

Model: log(fT3) ~ habitat location + age group					
Predictors	Estimates	SE	CI	Statistic	*P* value
Intercept	3.23	0.17	2.89 to 3.56	19.14	**<0.001**
Study site (Samburu National Reserve)	0.70	0.22	0.26 to 1.14	3.15	**0.002**
Study site (Agropastoral)	2.07	0.21	1.65 to 2.49	9.82	**<0.001**
Age group (juvenile)	0.23	0.22	−0.20 to 0.66	1.05	0.294
Age group (subadult)	0.16	0.20	−0.23 to 0.55	0.79	0.429
Observations	116				
*R* ^2^/*R*^2^ adjusted	0.53/0.51				

The reference category for the study site is the Lower Imenti Forest, while the reference category for the age group is adult. Variables that were statistically significant were indicated in bold.

## Discussion

We assessed the physiological stress response (as measured by fGCM concentrations) and nutritional stress response (as measured by fT3 concentrations) of African elephants within the Lower Imenti forest, an area that had experienced habitat degradation and subsequent invasion by *L. camara*. We compared fT3 and fGCM levels in elephant faecal samples collected from the Lower Imenti forest to those collected from individuals within Samburu National Reserve during the severe 2022 drought that resulted in high elephant mortality. We also compared hormone levels from Imenti samples to those from individuals within an agropastoral area in a nearby ecosystem where human–elephant conflict is prevalent, resulting in high physiological stress. We found lower concentrations of fT3 and higher concentrations of fGCM among individuals within the Lower Imenti forest compared to individuals within the Samburu National Reserve or agropastoral area, suggesting the sampled Imenti elephants were experiencing a higher nutritional stress response than that of the Samburu elephants during a severe drought, and a greater physiological stress response than elephants in a human–elephant conflict zone. We found no effect of age group on fT3 or fGCM concentrations, suggesting nutritional and adrenal stress were not significantly different according to age. Our findings provide insights into how degraded habitats, particularly those invaded by invasive plant species such as *L. camara,* influence the nutritional and physiological responses of African elephants, which will be critical in initiating habitat restoration efforts and other conservation interventions.

The lower fT3 concentrations from faecal samples found in the Lower Imenti Forest Reserve during a typical dry season were striking, given that the samples from Samburu were from elephants experiencing drought-related mortality. The extremely low fT3 concentrations among individuals in Lower Imenti compared to individuals within Samburu could have been attributed to two factors. First, historical habitat degradation in the Imenti study site likely facilitated the invasion of *L. camara,* which is toxic to and generally not eaten by elephants (Sharma *et al.*, 2007; Wilson *et al.*, 2013; Wilson *et al.*, 2014). The invasive plant reduces and displaces native, palatable vegetation, limiting access to alternative food sources during the dry season when forage quality and quantity are already low (Sundaram and Hiremath, 2012). In addition, *L. camara* suppresses native species that elephants may forage on by forming dense, impenetrable stands and disrupting the physical structure of the habitat (Wilson *et al.*, 2014; Shackleton *et al.*, 2017). In other studies, thyroid hormone levels have been associated with food limitation, decreasing during periods of energy restriction and increasing when energy is abundant (Behringer *et al.*, 2018). In contrast, elephant faecal samples collected within agropastoral areas, where elephants engage in crop raiding, had higher fT3 concentrations compared to other sites. fT3 concentrations have been reported to positively correlate with forage availability (measured by the normalized difference vegetation index [NDVI]) among free-ranging African elephants in the Madikwe Reserve, South Africa (Szott *et al.*, 2020). Cultivated vegetation at agropastoral sites offers higher nitrogen and digestible energy than natural vegetation (Pokharel *et al.*, 2019), which may explain the elevated fT3 concentrations observed in elephants inhabiting these areas. Higher fT3 concentrations have also been observed in maned wolves (*Chrysocyon brachyurus*) utilizing agricultural areas during peak harvest activity (Vynne *et al.*, 2014) and in African elephants found within agropastoral areas compared to other land use types (Oduor *et al.*, 2024). Elephants preferred utilizing agricultural areas during the brown-down stages because the crops contained significantly higher digestible energy (Branco *et al.*, 2019), which likely resulted in the higher fT3 concentrations. Although elephants within the Samburu National Reserve were experiencing a prolonged drought, which resulted in the death of 70 elephants within the ecosystem (WRTI, 2022), we expected elephant faecal samples within the area to have lower fT3 concentrations compared to those from the Lower Imenti Forest Reserve, given the starvation-induced mortality observed in the system (Flier *et al.*, 2000). However, contrary to our expectations, elephant faecal samples collected from the Lower Imenti Forest had lower fT3 concentrations compared to those from the Samburu National Reserve or agropastoral areas. We suspect the low fT3 concentrations among elephants in the Lower Imenti forest reflected their poor diets resulting from a lack of appropriate forage on account of the highly degraded nature of the ecosystem. However, we were not able to observe foraging patterns or analyse faecal nutrient content in the dung to assess the actual quality of their diet.

Secondly, given that elephants are highly mobile and generalist herbivores, it is surprising that they would be impacted to this degree by *L. camara* invasion rather than simply move to a different area. At the time of this study, a newly erected electric fence had curtailed the movement of elephants from the forest to Mt. Kenya, and a one-way gate only allowed elephants to enter the fenced area from the agricultural side, but prevented them from exiting (Ikime *et al.*, 2023). It is possible that this recent fencing installation inhibited the movements of the study elephants, due to a lack of familiarity with the access point or confusion by their normal seasonal movements being blocked. As a result, they appeared to be stuck in the lower Imenti section of the forest.

While the usefulness of GC levels as proxy indicators of stress and welfare has been critically evaluated, the assumption that elevated GC levels indicate poor welfare remains widespread (Mormède *et al.*, 2007; Veissier and Boissy, 2007; Ralph and Tilbrook, 2016). We found higher fGCM concentrations in faecal samples from elephants in the Lower Imenti Forest compared to those from elephants in agropastoral areas or Samburu. In agropastoral areas, elephants frequently experience high levels of human–elephant conflict, prompting farmers to employ various deterrent methods such as firecrackers, homemade firearms or small-calibre weapons (Nyhus and Sumianto, 2000; Gunaryadi *et al.*, 2017). These interactions are expected to elevate stress levels in elephants, reflected by higher fGCM concentrations (Ahlering *et al.*, 2011). We therefore expected to observe higher fGCM concentrations in elephant faecal samples collected in agropastoral areas compared to those from the Lower Imenti Forest or Samburu, where overt human–elephant conflict is not as common. Thus, it was surprising that our results showed the opposite: despite the dynamicity in physiological reactivity (Pokharel and Brown, 2023), elephants in the Lower Imenti Forest exhibited higher fGCM concentrations than those in agropastoral areas or Samburu National Reserve. It is noted that Samburu National Reserve serves as a critical refuge from human disturbance, and the elephants tend to exhibit calm behaviour and habituation to vehicle presence, despite past poaching pressures (Goldenberg *et al.*, 2017).

We attributed the higher fGCM concentrations in the Lower Imenti Forest to two main factors. First, we believed spatial restriction impacted the physiology and fGCM levels observed in the Imenti elephants. The spatial refuge hypothesis, as proposed by Jachowski *et al.* (2012, 2013), suggests that animals may voluntarily limit their movements to perceived refuges in response to stress, often resulting in elevated fGCM concentrations. In contrast, in this study, the restricted mobility of elephants in the Lower Imenti Forest is not due to active refuge seeking but externally imposed limitations. Specifically, the one-way gate that prevents access to Mt. Kenya confined elephants to an area of only 128 km^2^ (Ikime *et al.*, 2023). Such spatial restriction may itself act as a stressor, contributing to the elevated fGCM levels observed. Similar patterns have been reported elsewhere, where elephants with smaller home ranges showed higher fGCM concentrations at Phinda Reserve in South Africa (Jachowski *et al.*, 2012) and resident elephants with limited ranging patterns had higher fGCM levels than partial migrants at Mpala Ranch, Laikipia County, Kenya (Oduor *et al.*, 2020). Conversely, elephants in Samburu and agropastoral areas of Laikipia County do not face similar movement restrictions, ranging up to 10 677 km^2^ (Kuria *et al.,* 2024).

Second, invasive plant species are linked to habitat changes, which may potentially affect food availability and predation levels for native wild animals (Stewart *et al.*, 2021). Studies have explored the relationship between habitat quality and fGCM concentrations in wildlife. For example, Horcajada-Sánchez *et al.* (2019) observed higher fGCM concentrations in roe deer (*Capreolus capreolus*) in areas with poor habitat quality at the Sierra de Guadarrama National Park in the central Iberian Peninsula. In free-ranging Asian elephants, higher habitat quality indicated by higher NDVI values and elevated faecal nitrogen levels, a proxy for diet quality, was associated with reduced fGCM concentrations (Pokharel *et al.*, 2019). In Madagascar, collared brown lemurs (*Eulemur collaris*) living in degraded forests exhibited significantly higher fGCM concentrations than those inhabiting conserved forests (Balestri *et al.*, 2014). The invasion of *L. camara* in the Lower Imenti forest, facilitated by slash-and-burn agricultural practices (KWS, 2000), may have adversely affected habitat quality, thereby contributing to elevated fGCM concentrations observed in this area relative to Samburu or the agropastoral area.

The lack of differences between fGCM and fT3 across age classes could be explained by the very poor conditions for all elephants. Generally, young elephants are more susceptible to ecological fluctuations in habitat quality (Wittemyer *et al.*, 2021), which could influence both fGCM and fT3 concentrations in comparison to older elephants. Studies in South Africa on African savanna elephants (Szott *et al.*, 2020) and on captive Asian elephants (*Elephas maximus*) (LaDue *et al.*, 2023) found higher fT3 concentrations in juveniles compared to adults. This was expected due to the action of thyroid hormone in controlling metabolism during the growing period of an animal. Low thyroid hormones can impair growth and development in young animals (Hulbert, 2000), and the lack of a difference in fT3 concentrations among different age categories in our study may indicate especially inadequate thyroid activity for juveniles who are still in a critical phase of growth. The relationship between fGCM concentrations and the age group of African elephants in other studies has, however, achieved mixed results. While some studies have found higher fGCM concentrations in free-ranging adult African elephants (Oduor *et al.*, 2020), others have found no relationship between fGCM concentrations and age group in free-ranging African elephants (Ganswindt *et al.*, 2005; Viljoen *et al.*, 2008). Although the Lower Imenti Forest Reserve was considered to be a staging ground for crop-raiding elephants in the area, the establishment of an electric fence and confinement of elephants in the area could have influenced both their physiological and metabolic states.

Our study was limited in several ways. First, we were unable to quantify the extent of the lantana invasion in the Imenti study site, beyond noting that it is extensive and is the dominant plant in the area. Second, we were unable to assess the diet of the elephants in Imenti to better relate their hormone levels directly to their diet. In addition, by collecting the samples exclusively during the dry season, we could not assess seasonal variation in fGCM and fT3 concentrations in Imenti. To account for this, we compared hormone levels from Imenti to those from samples collected during the dry season at our other sites. Further studies on dietary quality and the consumption of *L. camara* species within the forest would clarify how diet influences their physiological and metabolic responses.

## Conclusions

Measuring the physiological response of an individual to a changing environment can yield insights into habitat quality and provide indicators of populations in trouble (Ellis *et al.*, 2012). Our study demonstrated that elephants inhabiting degraded habitats that had undergone major changes in vegetation types through invasion by *L. camara* and are restricted by an electric fence exhibited both elevated fGCM and reduced fT3 concentrations, suggesting heightened physiological stress and compromised metabolic function. These findings highlight how habitat degradation and barriers to natural movement can act together to disrupt the endocrine response of elephants. The feedback between habitat degradation and invasive species potentially has implications for disease prevalence as well. In our study, higher tick loads were found in carcasses of the Imenti forest elephants, which was of concern for the Kenya Wildlife Service given issues around tick-borne diseases.

Endocrine monitoring can be used to address the ubiquitous and impending challenges facing biodiversity (Homyack, 2010). Our findings demonstrate the value of using endocrine monitoring to determine the effect of habitat alteration and invasive species on wildlife physiology, which will be important in guiding conservation managers in developing habitat restoration efforts and making wildlife management decisions in the face of rapid anthropogenic land-use change (Wikelski and Cooke, 2006; Cooke and Suski, 2008; Hing *et al.*, 2016). The control of invasive species can be an effective intervention for the management of the Lower Imenti Forest Reserve in the long term.

## Supplementary Material

Web_Material_coaf060

## Data Availability

Due to the endangered conservation status of the African elephants, data will be shared upon request to the corresponding author.
